# Crystalline Phase-Dependent Emissivity of MoSi_2_ Nanomembranes for Extreme Ultraviolet Pellicle Applications

**DOI:** 10.3390/nano15191488

**Published:** 2025-09-29

**Authors:** Haneul Kim, Young Woo Kang, Jungyeon Kim, Taeho Lee, Jinho Ahn

**Affiliations:** 1 Division of Materials Science and Engineering, Hanyang University, Seoul 04763, Republic of Korea; milky-way@nate.com (H.K.); kyw9412@naver.com (Y.W.K.); lbhxlch@naver.com (J.K.); 2 CH^3^IPS Innovation Research Center, Hanyang University, Seoul 04763, Republic of Korea; 3 EUV-IUCC (Industry University Cooperation Center), Hanyang University, Seoul 04763, Republic of Korea; taeholee@hanyang.ac.kr

**Keywords:** extreme ultraviolet, pellicle, molybdenum disilicide, crystal structure, carrier density, emissivity

## Abstract

Extreme ultraviolet (EUV) pellicles must withstand intense thermal stress during exposure due to their limited heat dissipation, which results from their ultrathin geometry and the vacuum environment within EUV scanners. To address this challenge, we investigated the crystalline phase-dependent emissivity of nanometer-thick molybdenum disilicide (MoSi_2_) membranes. Membranes exhibiting amorphous, hexagonal, and tetragonal phases were independently prepared via controlled annealing, and their thermal radiation properties were evaluated using heat-load testing under emulated EUV scanner conditions. The Hall effect measurements revealed distinct variations in carrier density and mobility across phases, which were theoretically correlated with emissivity using the Lorentz–Drude model. The results demonstrate that emissivity increases in the hexagonal phase due to increased carrier density and reduced scattering, offering improved thermal radiation performance. These findings establish the phase engineering of conductive silicides as a viable strategy for enhancing radiative cooling in EUV pellicles and offer a theoretical framework applicable to other high-temperature nanomaterials.

## 1. Introduction

The scaling limitations of 193 nm ArF lithography led to the development of next-generation lithography technologies employing a 13.5 nm wavelength light source, referred to as extreme ultraviolet (EUV) lithography. With the implementation of EUV lithography in high-volume semiconductor manufacturing at technology nodes of 5 nm or smaller, effective contamination control within EUV scanners becomes crucial to ensuring high yield [1,2]. One key strategy for achieving such contamination control is EUV pellicle implementation. An EUV pellicle is a cover membrane that protects the EUV mask from contamination during exposure. It must be ultrathin—only tens of nanometers thick—to minimize throughput losses while maintaining stability under extreme conditions, posing significant technical challenges [3,4]. Additionally, during exposure, EUV radiation absorption increases the temperature of the pellicle membrane, resulting in thermal stress that can cause structural damage and wrinkling, leading to potential image distortion and membrane failure [5]. This phenomenon indicates the need to effectively cool the pellicle during lithography processes.

Heat dissipation in materials is primarily mediated via conduction, convection, and radiation [6]. However, the nanoscale thickness of the pellicle limits conductive heat transfer, and the high-vacuum environment within the EUV scanner considerably suppresses convective cooling, leaving radiative heat transfer as the dominant thermal dissipation pathway [7]. To improve radiation efficiency, previous studies have explored high-emissivity materials, including Ru, metal silicides, and metal carbides [8,9,10]. In particular, molybdenum disilicide (MoSi_2_) has been identified as a promising EUV pellicle material due to its high EUV transmittance, low reflectance, and excellent mechanical strength [11]. As the thermal properties of MoSi_2_ can be influenced by its crystalline phase, evaluating the efficiency of radiative cooling across different phases, including the amorphous, hexagonal, and tetragonal phases, is crucial for its effective implementation in EUV pellicle applications [12,13].

In this study, the influence of the crystalline phase of MoSi_2_ on its thermal radiation efficiency was systematically investigated. Phase-dependent and phase-induced variations in charge carrier density were quantitatively analyzed to evaluate their impacts on emissivity. The observed relationship was then theoretically validated using the Lorentz–Drude model, confirming the underlying mechanism driving changes in emissivity. Based on the results, the optimal crystalline phase for maximizing the thermal emissivity of MoSi_2_-based EUV pellicles is proposed.

## 2. Materials and Methods

### 2.1. Sample Fabrication and Analysis

**Figure 1:**
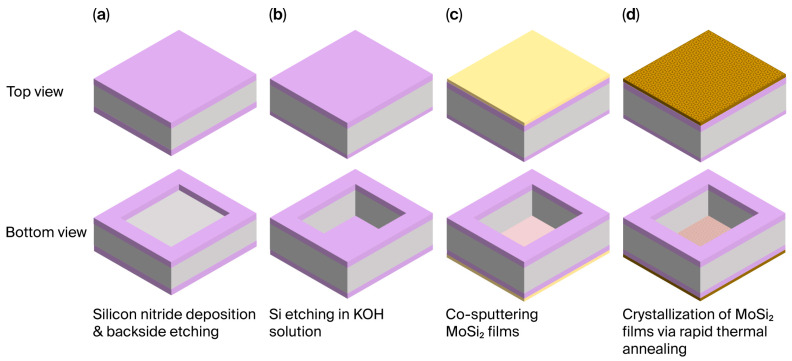
The schematics of the molybdenum disilicide (MoSi_2_)/silicon nitride (SiN_x_) pellicle composite fabrication process: (**a**) SiN_x_ deposition and backside patterning via reactive-ion etching; (**b**) SiN_x_ free-standing membrane fabrication via potassium hydroxide (KOH) wet etching; (**c**) MoSi_2_ film deposition via the co-sputtering method; (**d**) rapid thermal annealing processes at 600 and 900 °C for crystallization.

The MoSi_2_/SiN_x_ pellicle composite fabrication process is illustrated in Figure 1. First, a 60 nm silicon nitride layer was deposited on a silicon wafer via low-pressure chemical vapor deposition at 820 °C using dichlorosilane (SiH_2_Cl_2_) and ammonia gas. Photolithography and reactive-ion etching were then employed to form a backside window. Subsequently, wet etching was carried out in a 30% potassium hydroxide solution at 60 °C, followed by a cleaning process, resulting in a 10 mm × 10 mm free-standing SiN_x_ membrane. The 20 nm MoSi_2_ layer was deposited onto the membrane using the co-sputtering method, with substrate heating performed at 400 °C to enhance the film density. The MoSi_2_/SiN_x_ composite pellicle was crystallized using the rapid thermal annealing (RTA) system at 600 and 900 °C.

In this study, transmission electron microscopy (TEM) was used to measure the thicknesses of MoSi_2_ films subjected to different annealing temperatures. The crystalline phase transitions of the thin MoSi_2_ films were characterized using X-ray diffraction (XRD). Based on the measured film thickness results, Hall effect measurements were employed to determine the resistivity, carrier density, and mobility of the films. For Hall effect measurements, five independent thin-film samples were prepared and measured for each annealing condition. Unless otherwise specified, the reported values represent the average across these five films, and the error bars indicate root-mean-square (RMS) deviations.

### 2.2. Heat-Load Test

**Figure 2:**
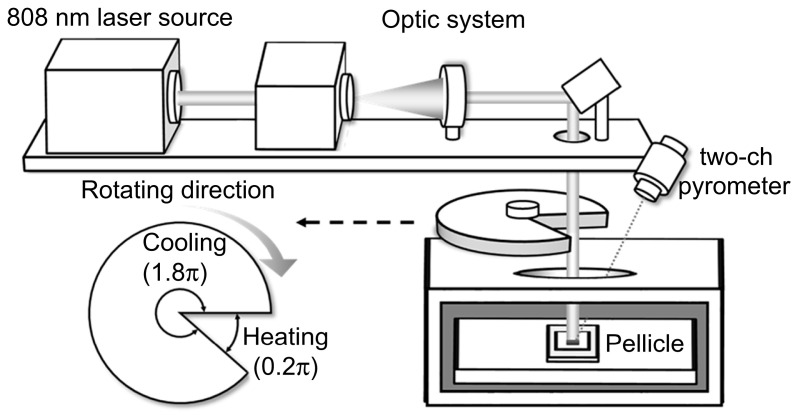
A schematic of the heat-load tester and rotating slit.

A heat-load tester, as illustrated in Figure 2, was used to evaluate the emissivity of the crystallized MoSi_2_/SiN_x_ pellicle composite. The equipment included a vacuum chamber that maintains a pressure below 4 × 10^−4^ Pa to replicate the EUV scanner environment. A rotating slit mechanism was incorporated to simulate a 1:9 scanning duty cycle.

Reference [7] reported on the temperature increase caused by absorption during EUV exposure, where the pellicle was uniformly irradiated using a 13.5 nm wavelength beam shaped into a curved rectangular slit pattern. To replicate this condition, a 7 mm × 7 mm square-shaped 808 nm laser was used to irradiate the pellicle, and the emissivity was calculated based on the absorbed heat energy. The absorption at 808 nm was measured using an ultraviolet–visible spectrometer, and the pellicle temperature was monitored using a two-channel pyrometer, which operated within a range of 400–1500 °C with a ±2% accuracy. For the heat-load experiments, five independent free-standing MoSi_2_/SiN_x_ membranes were measured under each annealing condition. The reported temperature and emissivity values represent the average of these five membranes, and the error bars correspond to RMS deviations.

## 3. Results and Discussion

### 3.1. MoSi_2_ Thin-Film Characteristics According to Annealing Temperature

**Figure 3:**
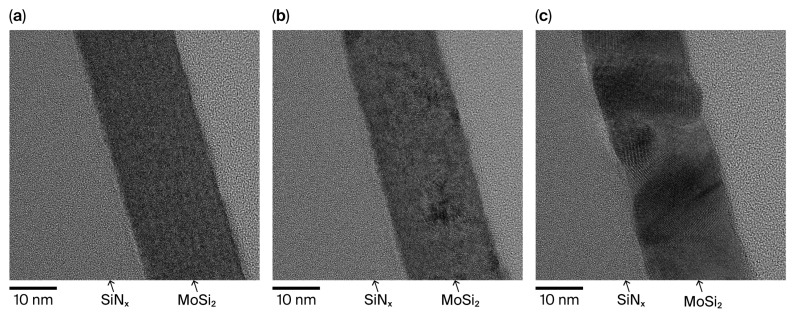
TEM cross-sectional images of the MoSi_2_/SiN_x_ pellicle composite: (**a**) as-deposited, (**b**) after annealing at 600 °C, and (**c**) after annealing at 900 °C.

**Figure 4:**
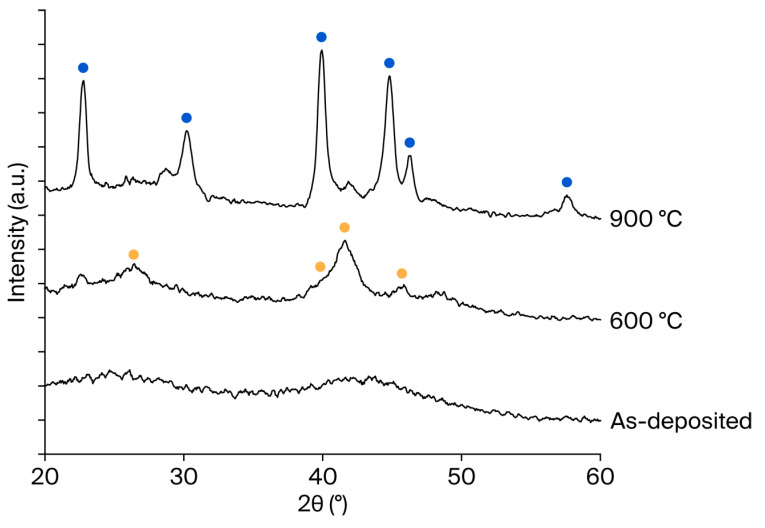
X-ray diffraction results for the MoSi_2_/SiN_x_ film as deposited and after annealing at 600 °C and 900 °C.

Figure 3 presents cross-sectional TEM images of the MoSi_2_/SiN_x_ films in the as-deposited state and after annealing at 600 and 900 °C. The film thickness remained constant at 20 nm under all annealing conditions, indicating that crystallization did not affect film thickness. Importantly, supplementary optical simulations confirm that a 20 nm MoSi_2_ membrane achieves >90% transmittance at 13.5 nm, verifying that the selected thickness is representative of application-level EUV pellicle requirements (Supplementary Figure S1). In the as-deposited state, the MoSi_2_ layer exhibited an amorphous structure. Upon annealing at 600 °C, nanometer-scale crystallites began to form, while annealing at 900 °C resulted in the growth of comparatively larger crystalline grains. To further quantify this observation, AFM measurements were conducted on three independent samples under each condition. The averaged root-mean-square roughness increased slightly from 0.576 nm (as-deposited) to 0.777 nm (600 °C) and 0.939 nm (900 °C), as summarized in Supplementary Figure S2. Although this increase reflects grain coarsening at increased temperature, the absolute roughness remains well below the EUV wavelength (13.5 nm), indicating a negligible contribution to additional scattering [14]. To identify the corresponding crystalline phases, XRD analysis was conducted. As shown in Figure 4, no distinct crystalline phase was observed in the as-deposited MoSi_2_ film. After annealing at 600 °C, the MoSi_2_ film exhibited a hexagonal phase, while annealing at 900 °C resulted in the formation of a tetragonal phase. In addition to XRD analysis, compositional characterization was conducted using XPS depth profiling. The results confirmed a uniform Mo:Si ratio close to 1:2 across the film depth, consistent with the expected stoichiometry of MoSi_2_ (Supplementary Figure S3)_._

### 3.2. Dependence of the MoSi_2_ Crystalline Phase on the Electrical Characteristics of Materials

The crystalline phase of a material intrinsically determines its band structure by defining the lattice symmetry and atomic arrangement. Phase transitions induce band structural reconstruction, which modifies the density of states and the bandgap. These electronic changes directly affect carrier density and activation, which subsequently influence the scattering mechanisms within the material. As emissivity is governed by both electron–electron and electron–phonon interactions, it is also sensitive to variations in carrier behavior [15,16].

**Figure 5:**
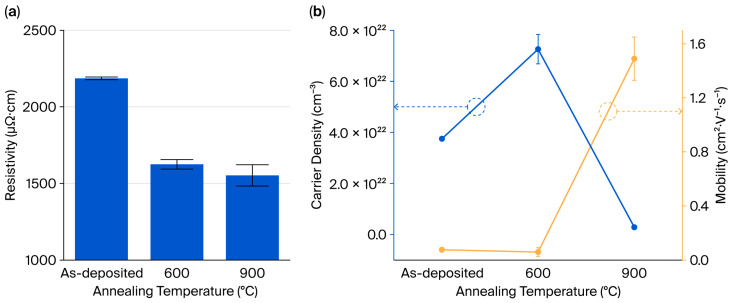
The (**a**) resistivity of the MoSi_2_ thin film and (**b**) carrier density and mobility through Hall measurements of the thin MoSi_2_ film as deposited and after annealing at 600 and 900 °C.

To investigate this relationship, Hall effect measurements were performed on the MoSi_2_ thin films subjected to different annealing temperatures. As shown in Figure 5a, the resistivity progressively decreased from 2186 (as-deposited) to 1625 (600 °C) and 1552 μΩ·cm (900 °C), indicating enhanced electrical conductivity at increasing annealing temperatures.

The origin of this trend was further investigated through carrier density and mobility measurements, as illustrated in Figure 5b. In the as-deposited amorphous state, electrons were identified as the majority carriers, with a carrier density of 3.75 × 10^22^ cm^−3^ and a mobility of 0.075 cm^2^/V·s. Although MoSi_2_ typically exhibits p-type behavior in its crystalline form, the presence of delocalized states and nanocrystallites in the amorphous phase could support electron-dominated conduction through various mechanisms such as hopping or multiband transport [17,18].

After annealing at 600 °C, a hexagonal phase was formed, and holes became the majority carrier type. The carrier density increased to 7.26 × 10^22^ cm^−3^, while mobility decreased slightly to 0.06 cm^2^/V·s. This behavior indicates enhanced carrier activation accompanied by increased carrier scattering.

Upon subsequent annealing at 900 °C, the tetragonal phase emerged. At this stage, the hole carrier density decreased dramatically to 2.85 × 10^21^ cm^−3^, while mobility increased considerably to 1.49 cm^2^/V·s. This behavior indicates a reduction in scattering due to the lower carrier concentration, allowing for more efficient charge transport, thus resulting in reduced resistivity.

### 3.3. Effects of Carrier Density on the Emissivity of Crystallized MoSi_2_

**Figure 6:**
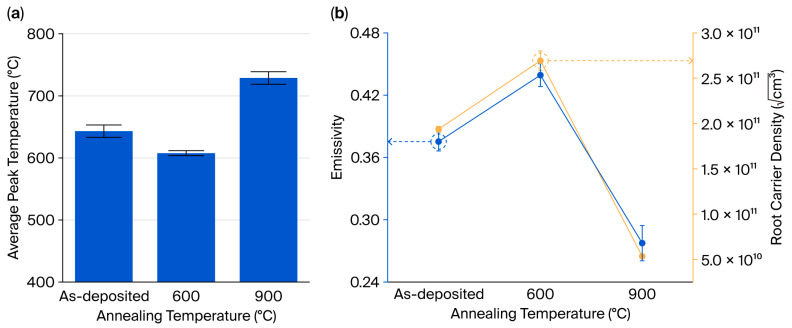
(**a**) Average peak temperature measured via the heat-load test. (**b**) A comparison of the calculated emissivity results and the square root of the carrier density of the MoSi_2_/SiN_x_ pellicle composite as deposited and after annealing at 600 and 900 °C.

Figure 6a shows the results of a heat-load test conducted on the MoSi_2_/SiN_x_ composite pellicle at an applied thermal power density of 1.5 W/cm^2^. The measured average peak temperatures for the as-deposited film and the films annealed at 600 and 900 °C were 643.1, 607.7, and 728.8 °C, respectively. These temperature values were subsequently used to calculate the emissivity based on the Stefan–Boltzmann law as follows [19]:

(1) $$\alpha \cdot P=\epsilon \cdot \sigma \cdot S\cdot \left(T^{4}-T_{s}^{4}\right),$$

where *α* is the material’s absorption coefficient, *P* is the incident light power, *ϵ* is the emissivity, *σ* is the Stefan–Boltzmann constant (5.67 × 10^−8^ W/m^2^K), *S* is the radiating area, *T* is the peak temperature, and *T*_s_ is the surrounding temperature.

Figure 6b shows the correlation between emissivity, derived using Equation (1), and the square root of the carrier density. The calculated emissivity values for the as-deposited film and the films annealed at 600 and 900 °C were 0.375, 0.439, and 0.277, respectively. A comparison with the square root of the measured carrier densities revealed a consistent trend, indicating a direct correlation between the square root of the carrier density and emissivity. To validate the theoretical basis of this relationship, additional analyses were conducted on the physical parameters that govern thermal emissivity.

At several hundred degrees Celsius, thermal radiation mainly occurs in the infrared region, as described by Planck’s law. Under steady-state conditions, Kirchhoff’s law states that the emissivity of a material is equal to its absorbance at a given wavelength. Absorbance is determined by the material’s optical transmittance and reflectance, both of which depend on the refractive index (n) and the extinction coefficient (k) [20,21].

These optical constants are associated with the material’s dielectric response, which varies with frequency, described by the complex permittivity. The Lorentz–Drude model provides a framework for expressing this frequency-dependent response as follows [22]:

(2) $$\varepsilon \left(\omega \right)=1+\varepsilon_{Drude}+\sum_{n}\varepsilon_{Lorentz},$$

In Equation (2), the optical responses in the ultraviolet and visible spectral regions are predominantly governed via interband transitions, which are described using the Lorentz oscillator terms. In contrast, the response in the infrared region is dominated by the intraband transitions of free carriers and is characterized using the Drude term. Given that MoSi_2_ is a conductive material with a relatively high carrier density, its optical response in the infrared region can be approximated using only the Drude term as follows:

(3) $$\varepsilon_{Drude}\left(\omega \right)=\varepsilon_{\infty }-\frac{\omega_{p}^{2}}{-\omega^{2}-i\frac{\omega }{\tau }},$$

where the plasma frequency $\omega_{p}$ is defined as

(4) $$\omega_{p}=\sqrt{\frac{ne^{2}}{\varepsilon_{0}m_{e}^{*}}},$$

where n is the carrier density, e is the elementary charge, $\varepsilon_{0}$ is the vacuum permittivity, and $m_{e}^{*}$ is the carrier effective mass.

The extinction coefficient k, which characterizes the imaginary component of the complex refractive index in the infrared region, can be approximated from the complex permittivity ($\varepsilon_{1}+i\varepsilon_{2}$) as follows [23]:

(5) $$k\approx \frac{1}{2}\left|\frac{\varepsilon_{2}}{\varepsilon_{1}}\right|\sqrt{\left|\varepsilon_{1}\right|},\;(\varepsilon_{1}>0),$$

(6) $$k\approx \sqrt{\left|\varepsilon_{1}\right|}\;\;(\varepsilon_{1}<0),$$

According to these relations, k is proportional to the square root of $\omega_{p}$, which, in turn, is proportional to the square root of the carrier density. As the emissivity is governed by k, it is directly affected by the carrier density.

The crystallization of MoSi_2_ modifies its band structure, thereby altering the carrier density and inducing a shift in the plasma frequency, as described in Equation (4). These shifts lead to variations in emissivity. It should be noted that the applicability of the Lorentz–Drude model to explain phase-dependent emissivity is particularly relevant for metal silicides and related intermetallic compounds, where phase transitions can substantially alter carrier density and electronic structure. In contrast, for pure metals with intrinsically high and relatively invariant carrier densities, phase transitions are expected to have only a minor impact on the free-carrier contribution; thus, the model’s explanatory power is limited. Nevertheless, uncertainties may arise from factors such as grain boundary scattering, effective mass assumptions, and the coexistence of multiple phases that should be considered when interpreting the quantitative accuracy of the model [9].

Consequently, the emissivity of MoSi_2_ thin films is phase-dependent and exhibits a strong correlation with the square root of the carrier density. These findings confirm that emissivity modulation originates from the structural phase transition and establish that the carrier density is a crucial design parameter for materials intended for use in high-temperature radiative environments.

## 4. Conclusions

In this study, the relationship between the carrier density and emissivity in MoSi_2_ nanomembranes was experimentally investigated as a function of the crystalline phase. The results were theoretically validated based on the Lorentz–Drude model. Phase transitions of the membrane film were induced through controlled annealing processes, and corresponding changes in their resistivity were observed. These resistivity variations were attributed to changes in carrier density and mobility within the MoSi_2_ thin films.

To experimentally verify the influence of carrier density on emissivity, heat-load tests were conducted under conditions designed to emulate the EUV scanner environment. Emissivity trends, analyzed within the Lorentz–Drude framework, confirmed the correlation between the electrical and radiative properties. Although the experiments were performed on coupon-sized membranes (10 mm × 10 mm) due to fabrication constraints, the observed trends represent an important step towards the development of full-size EUV pellicle. When scaled to full-size pellicles, however, additional factors such as stress distribution across the membrane, interfacial mismatch with supporting border, and fabrication-induced defects may influence emissivity. These aspects highlight the need for further investigation to establish scalability and reproducibility under practical manufacturing conditions.

These findings demonstrate that the hexagonal phase in MoSi_2_ enhances emissivity, thereby improving the thermal radiation efficiency and ensuring greater thermal stability in EUV pellicle applications. Furthermore, the theoretical framework established in this study can be extended to other metal silicide materials, highlighting its broader applicability.

Compared with conventional pellicle materials such as SiN_x_, Ru, and metal carbides, phase-engineered MoSi_2_ membranes provide enhanced radiative cooling efficiency while maintaining high EUV transmittance. Nevertheless, challenges remain with regard to oxidation resistance, hydrogen radical durability, and scalability for full-size pellicle fabrication, warranting further study. 

## Data Availability

The data that support the findings of this study, including the minimal dataset underlying the figures and tables, are available from the corresponding author upon reasonable request due to confidentiality obligations associated with ongoing industry collaboration and pending intellectual property considerations. The raw experimental files (e.g., XRD spectra, TEM DM3 images, heat-load test logs) are likewise available on request.

## Supplementary Materials

The following supporting information can be downloaded at: https://www.mdpi.com/article/10.3390/nano15191488/s1, Figure S1. Simulated EUV transmittance of MoSi_2_ membranes at a wavelength of 13.5 nm as a function of film thickness, obtained using the PROLITH simulation tool. For the optical constants of MoSi_2_ at the EUV wavelength, a refractive index of n = 0.9693 and an extinction coefficient of k = 0.0043 were used, as reported in the CXRO database. The results indicate that a 20 nm MoSi_2_ membrane achieves >90% transmittance, verifying that the selected thickness is representative of application-level EUV pellicle requirements. Figure S2. Representative AFM 3D surface images of MoSi_2_ thin film for different annealing conditions: (a) as-deposited (Rq = 0.576 ± 0.31 nm), (b) 600 °C annealed (Rq = 0.778 ± 0.39 nm), and (c) 900 °C annealed (Rq = 0.939 ± 0.11 nm). Roughness values were obtained as averages from three separate samples for each condition, and the images shown correspond to representative measurements. Figure S3. XPS depth profile of MoSi_2_ thin films. A very thin surface oxidation layer with sub-nm thickness is observed at the outermost surface due to ambient exposure. Beyond this, the composition rapidly stabilizes to a Mo:Si ratio of approximately 1:2, confirming that the film maintains the expected stoichiometry of MoSi_2_ throughout its depth.

## Abbreviations

The following abbreviations are used in this manuscript:

EUV	Extreme ultraviolet
MoSi_2_	Molybdenum disilicide
KOH	Potassium hydroxide
TEM	Transmission electron microscopy
XRD	X-ray diffraction
