# Supplementary material for: Crystalline Phase-Dependent Emissivity of MoSi2 Nanomembranes for Extreme Ultraviolet Pellicle Applications
Source: Nanomaterials (Basel). 2025 Sep 29;15(19):1488. doi: 10.3390/nano15191488 (PMC12526329; doi:10.3390/nano15191488)
Supplement: Supplementary file 1 [file nanomaterials-15-01488-s001.zip › nanomaterials-3869930-supplementary.pdf]

Supporting Information for

Crystalline Phase-Dependent Emissivity of $MoSi_2$ Nanomembranes for Extreme Ultraviolet Pellicle Applications

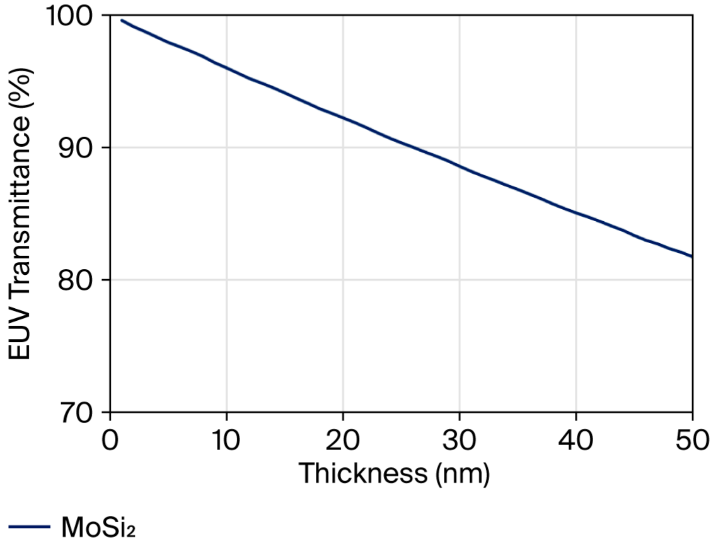

Figure S1. Simulated EUV transmittance of $MoSi_2$ membranes at a wavelength of 13.5 nm as a function of film thickness, obtained using the PROLITH simulation tool. For the optical constants of $MoSi_2$ at the EUV wavelength, a refractive index of n = 0.9693 and an extinction coefficient of k = 0.0043 were used, as reported in the CXRO database. The results indicate that a 20 nm $MoSi_2$ membrane achieves >90% transmittance, verifying that the selected thickness is representative of application-level EUV pellicle requirements.

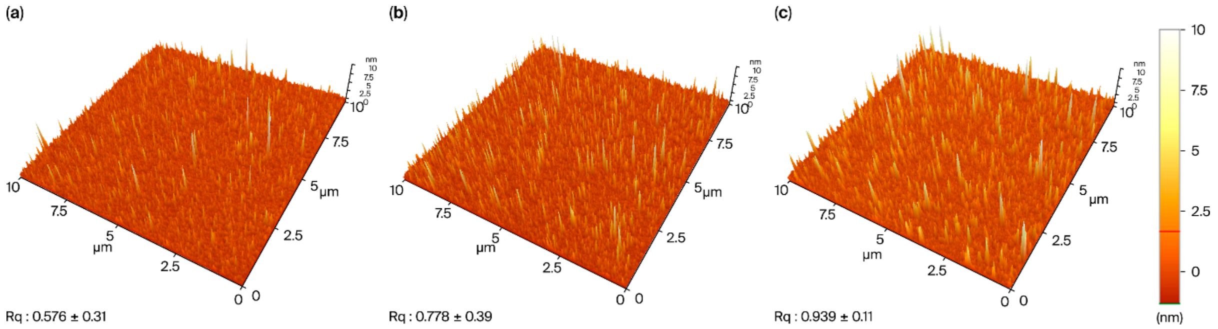

Figure S2. Representative AFM 3D surface images of $MoSi_2$ thin film for different annealing conditions: (a) as-deposited (Rq = 0.576 ± 0.31 nm), (b) 600 °C annealed (Rq = 0.778 ± 0.39 nm), and (c) 900 °C annealed (Rq = 0.939 ± 0.11 nm). Roughness values were obtained as averages from three separate samples for each condition, and the images shown correspond to representative measurements.

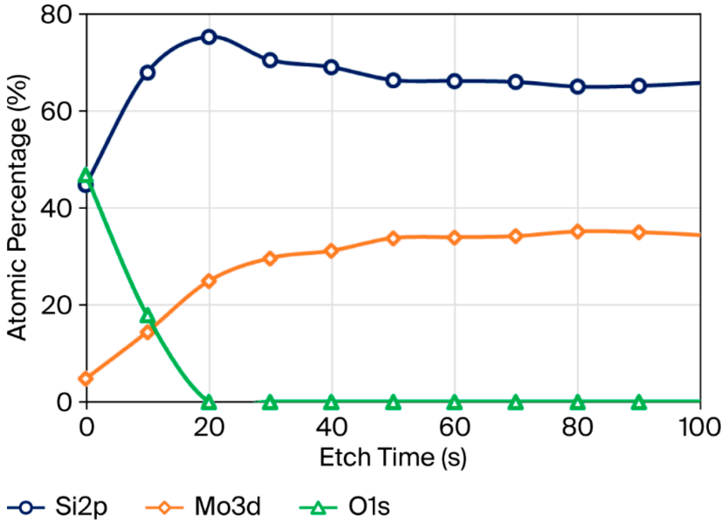

Figure S3. XPS depth profile of $MoSi_2$ thin films. A very thin surface oxidation layer with sub-nm thickness is observed at the outermost surface due to ambient exposure. Beyond this, the composition rapidly stabilizes to a Mo:Si ratio of approximately 1:2, confirming that the film maintains the expected stoichiometry of $MoSi_2$ throughout its depth.
